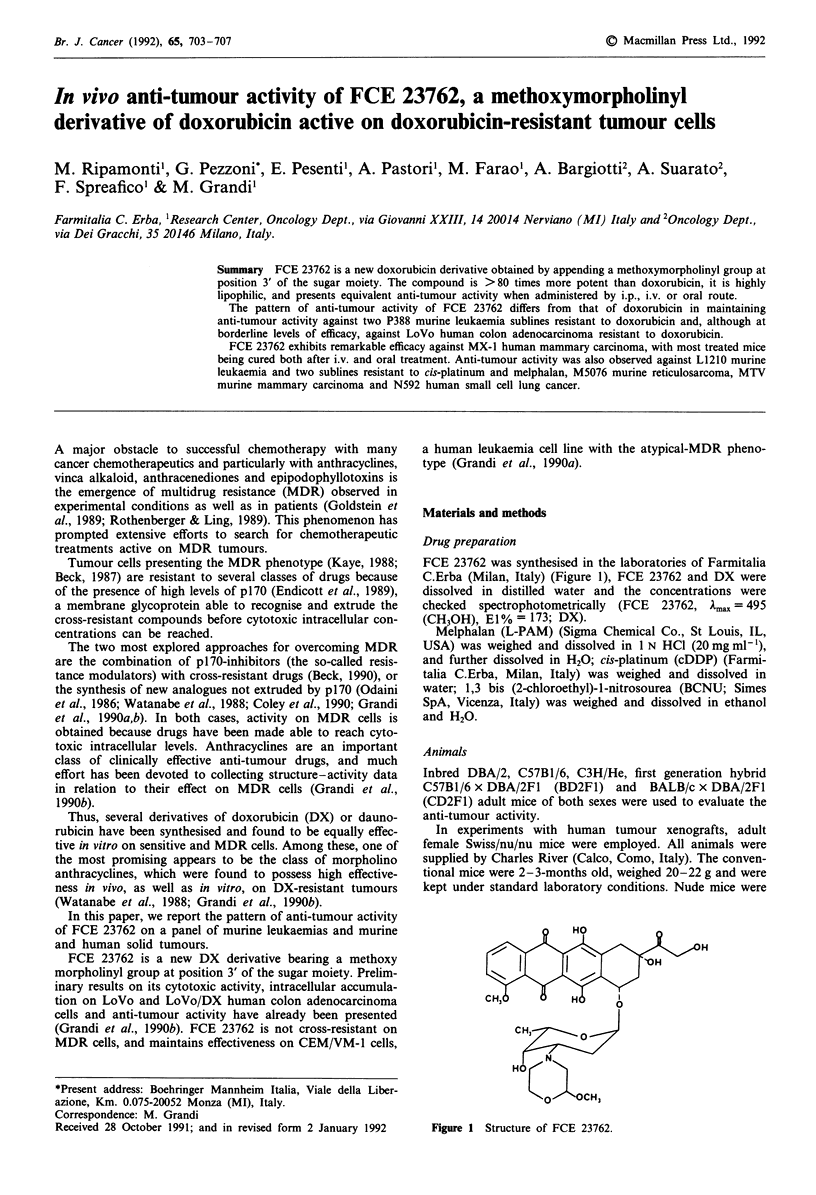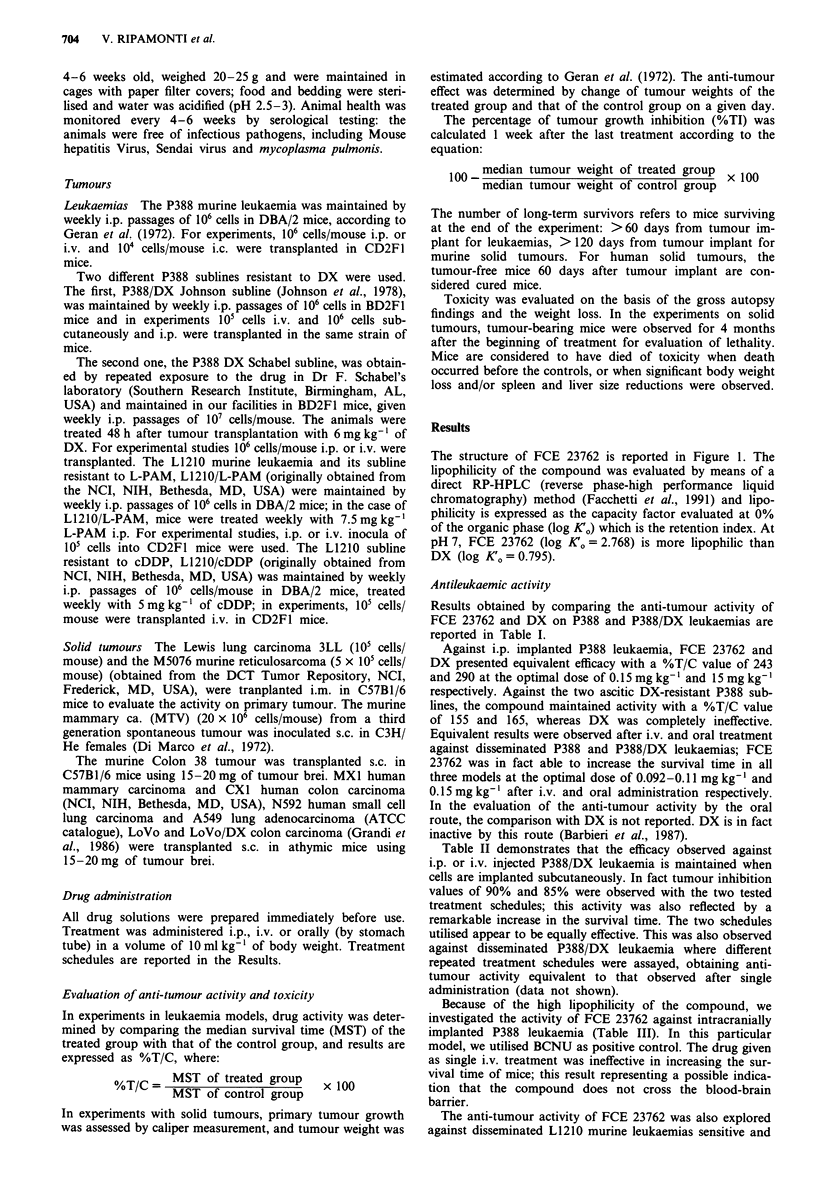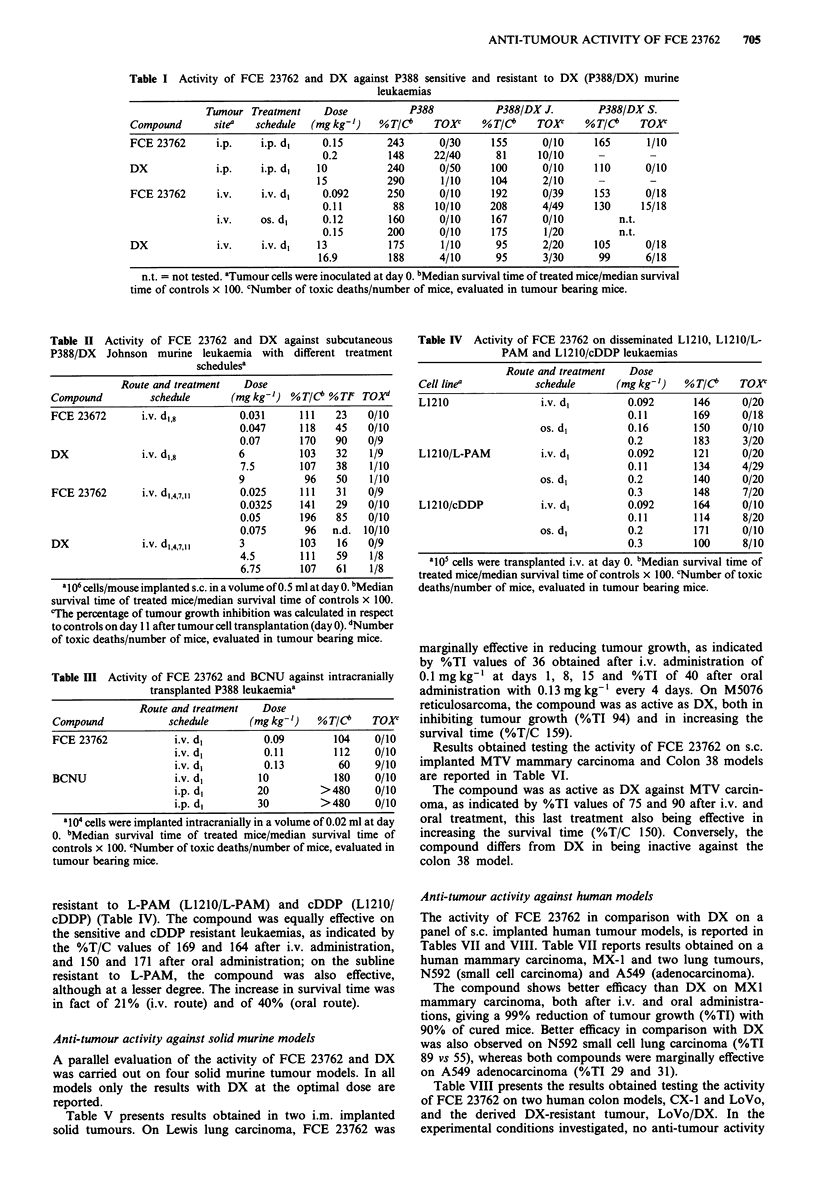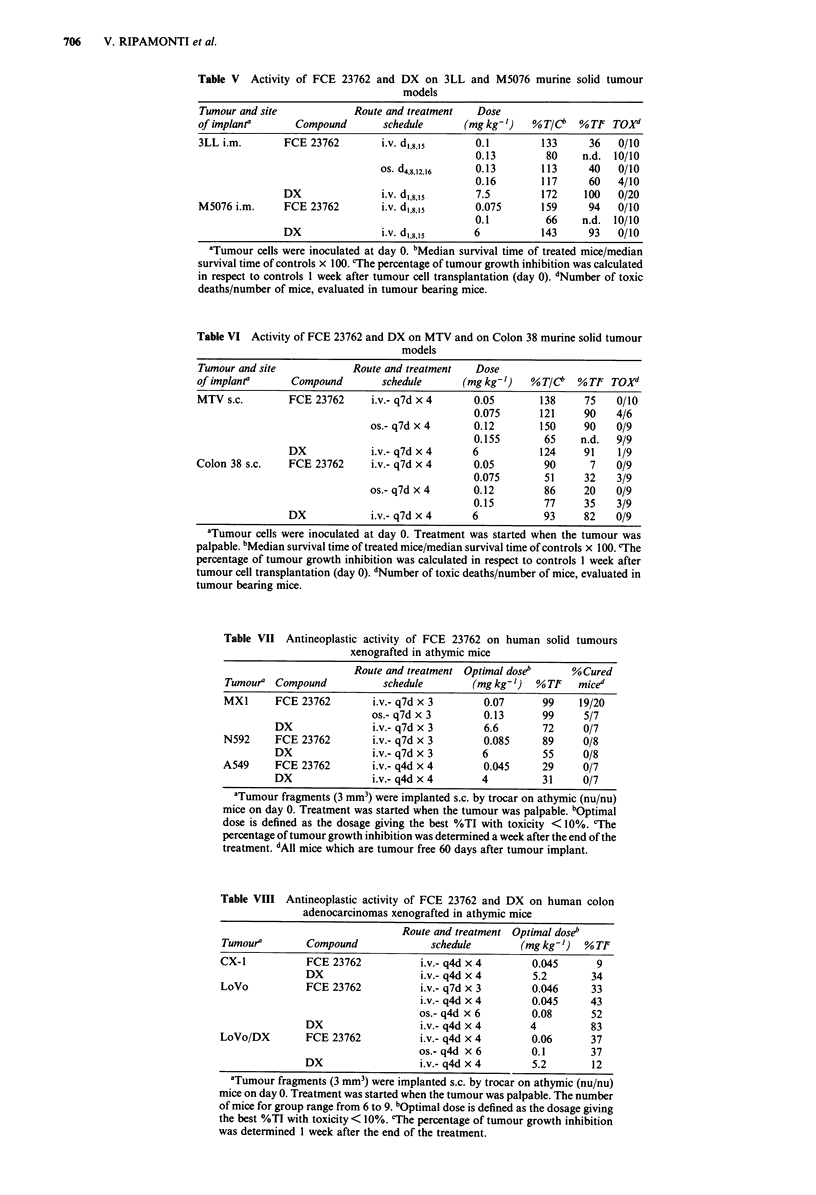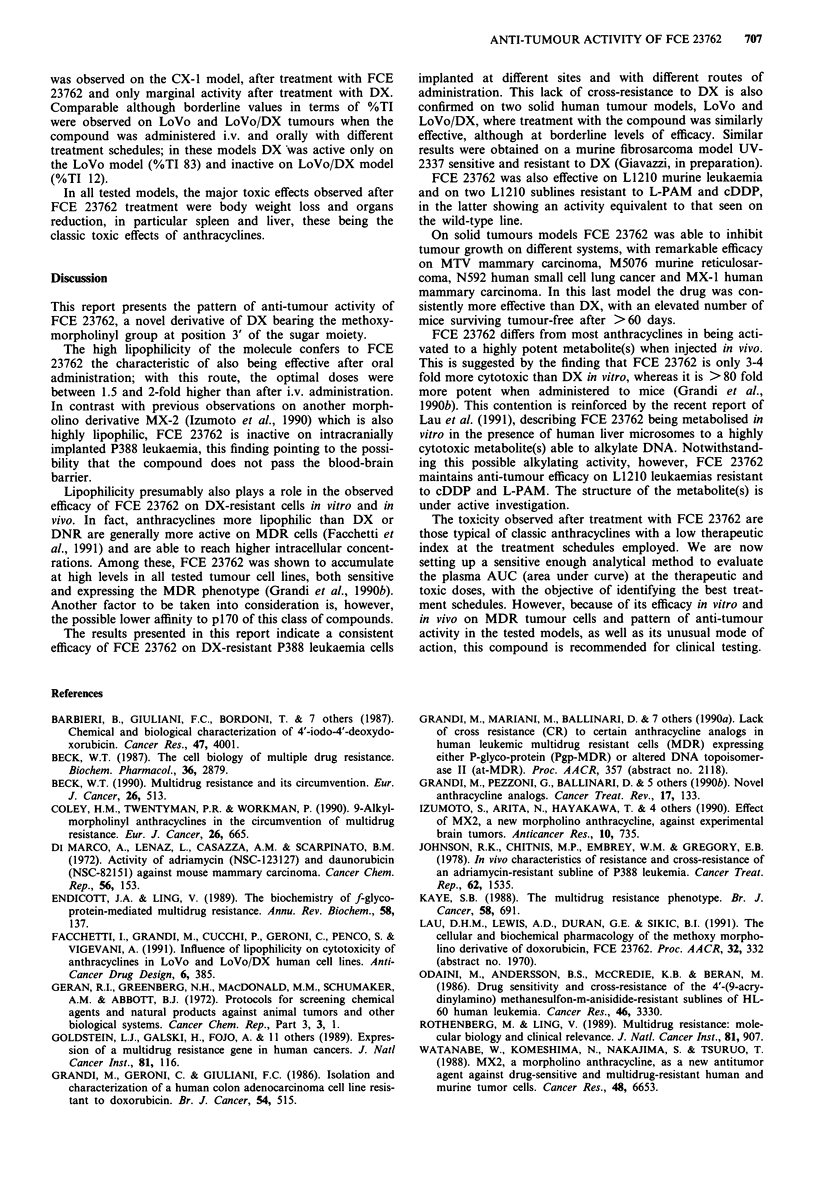# In vivo anti-tumour activity of FCE 23762, a methoxymorpholinyl derivative of doxorubicin active on doxorubicin-resistant tumour cells.

**DOI:** 10.1038/bjc.1992.148

**Published:** 1992-05

**Authors:** M. Ripamonti, G. Pezzoni, E. Pesenti, A. Pastori, M. Farao, A. Bargiotti, A. Suarato, F. Spreafico, M. Grandi

**Affiliations:** Research Center, Oncology Dept., Nerviano (MI), Italy.

## Abstract

FCE 23762 is a new doxorubicin derivative obtained by appending a methoxymorpholinyl group at position 3' of the sugar moiety. The compound is greater than 80 times more potent than doxorubicin, it is highly lipophilic, and presents equivalent anti-tumour activity when administered by i.p., i.v. or oral route. The pattern of anti-tumour activity of FCE 23762 differs from that of doxorubicin in maintaining anti-tumour activity against two P388 murine leukaemia sublines resistant to doxorubicin and, although at borderline levels of efficacy, against LoVo human colon adenocarcinoma resistant to doxorubicin. FCE 23762 exhibits remarkable efficacy against MX-1 human mammary carcinoma, with most treated mice being cured both after i.v. and oral treatment. Anti-tumour activity was also observed against L1210 murine leukaemia and two sublines resistant to cis-platinum and melphalan, M5076 murine reticulosarcoma, MTV murine mammary carcinoma and N592 human small cell lung cancer.


Br. J. Cancer (1992), 65, 703-707 © Macmillan Press Ltd., 1992

### *In vivo* anti-tumour activity of FCE 23762, a methoxymorpholinyl derivative of doxorubicin active on doxorubicin-resistant tumour cells

M. Ripamonti[1], G. Pezzoni*, E. Pesenti[1], A. Pastori[1], M. Farao[1], A. Bargiotti[2], A. Suarato[2], F. Spreafico[1] & M. Grandi[1]

*Farmitalia C. Erba, [1]Research Center, Oncology Dept., via Giovanni XXIII, 14 20014 Nerviano (MI) Italy and [2]Oncology Dept., via Dei Gracchi, 35 20146 Milano, Italy.*

**Summary** FCE 23762 is a new doxorubicin derivative obtained by appending a methoxymorpholinyl group at position 3' of the sugar moiety. The compound is >80 times more potent than doxorubicin, it is highly lipophilic, and presents equivalent anti-tumour activity when administered by i.p., i.v. or oral route.

The pattern of anti-tumour activity of FCE 23762 differs from that of doxorubicin in maintaining anti-tumour activity against two P388 murine leukaemia sublines resistant to doxorubicin and, although at borderline levels of efficacy, against LoVo human colon adenocarcinoma resistant to doxorubicin.

FCE 23762 exhibits remarkable efficacy against MX-1 human mammary carcinoma, with most treated mice being cured both after i.v. and oral treatment. Anti-tumour activity was also observed against L1210 murine leukaemia and two sublines resistant to *cis*-platinum and melphalan, M5076 murine reticulosarcoma, MTV murine mammary carcinoma and N592 human small cell lung cancer.

A major obstacle to successful chemotherapy with many cancer chemotherapeutics and particularly with anthracyclines, vinca alkaloid, anthracenediones and epipodophyllotoxins is the emergence of multidrug resistance (MDR) observed in experimental conditions as well as in patients (Goldstein *et al.*, 1989; Rothenberger & Ling, 1989). This phenomenon has prompted extensive efforts to search for chemotherapeutic treatments active on MDR tumours.

Tumour cells presenting the MDR phenotype (Kaye, 1988; Beck, 1987) are resistant to several classes of drugs because of the presence of high levels of p170 (Endicott *et al.*, 1989), a membrane glycoprotein able to recognise and extrude the cross-resistant compounds before cytotoxic intracellular concentrations can be reached.

The two most explored approaches for overcoming MDR are the combination of p170-inhibitors (the so-called resistance modulators) with cross-resistant drugs (Beck, 1990), or the synthesis of new analogues not extruded by p170 (Odaini *et al.*, 1986; Watanabe *et al.*, 1988; Coley *et al.*, 1990; Grandi *et al.*, 1990*a,b*). In both cases, activity on MDR cells is obtained because drugs have been made able to reach cytotoxic intracellular levels. Anthracyclines are an important class of clinically effective anti-tumour drugs, and much effort has been devoted to collecting structure-activity data in relation to their effect on MDR cells (Grandi *et al.*, 1990*b*).

Thus, several derivatives of doxorubicin (DX) or daunorubicin have been synthesised and found to be equally effective *in vitro* on sensitive and MDR cells. Among these, one of the most promising appears to be the class of morpholino anthracyclines, which were found to possess high effectiveness *in vivo*, as well as *in vitro*, on DX-resistant tumours (Watanabe *et al.*, 1988; Grandi *et al.*, 1990*b*).

In this paper, we report the pattern of anti-tumour activity of FCE 23762 on a panel of murine leukaemias and murine and human solid tumours.

FCE 23762 is a new DX derivative bearing a methoxy morpholinyl group at position 3' of the sugar moiety. Preliminary results on its cytotoxic activity, intracellular accumulation on LoVo and LoVo/DX human colon adenocarcinoma cells and anti-tumour activity have already been presented (Grandi *et al.*, 1990*b*). FCE 23762 is not cross-resistant on MDR cells, and maintains effectiveness on CEM/VM-1 cells, a human leukaemia cell line with the atypical-MDR phenotype (Grandi *et al.*, 1990*a*).

### Materials and methods

### *Drug preparation*

FCE 23762 was synthesised in the laboratories of Farmitalia C.Erba (Milan, Italy) (Figure 1), FCE 23762 and DX were dissolved in distilled water and the concentrations were checked spectrophotometrically (FCE 23762, $\lambda_{max} = 495$ ($CH_3OH$), E1% = 173; DX).

Melphalan (L-PAM) (Sigma Chemical Co., St Louis, IL, USA) was weighed and dissolved in 1 N HCl (20 mg ml$^{-1}$), and further dissolved in $H_2O$; *cis*-platinum (cDDP) (Farmitalia C.Erba, Milan, Italy) was weighed and dissolved in water; 1,3 bis (2-chloroethyl)-1-nitrosourea (BCNU; Simes SpA, Vicenza, Italy) was weighed and dissolved in ethanol and $H_2O$.

### *Animals*

Inbred DBA/2, C57Bl/6, C3H/He, first generation hybrid C57Bl/6 x DBA/2F1 (BD2F1) and BALB/c x DBA/2F1 (CD2F1) adult mice of both sexes were used to evaluate the anti-tumour activity.

In experiments with human tumour xenografts, adult female Swiss/nu/nu mice were employed. All animals were supplied by Charles River (Calco, Como, Italy). The conventional mice were 2-3-months old, weighed 20-22 g and were kept under standard laboratory conditions. Nude mice were

**Figure 1** Structure of FCE 23762.

*Present address: Boehringer Mannheim Italia, Viale della Liberazione, Km. 0.075-20052 Monza (MI), Italy.
Correspondence: M. Grandi
Received 28 October 1991; and in revised form 2 January 1992

4–6 weeks old, weighed 20–25 g and were maintained in cages with paper filter covers; food and bedding were sterilised and water was acidified (pH 2.5–3). Animal health was monitored every 4–6 weeks by serological testing: the animals were free of infectious pathogens, including Mouse hepatitis Virus, Sendai virus and *mycoplasma pulmonis*.

### Tumours

*Leukaemias* The P388 murine leukaemia was maintained by weekly i.p. passages of $10^6$ cells in DBA/2 mice, according to Geran *et al.* (1972). For experiments, $10^6$ cells/mouse i.p. or i.v. and $10^4$ cells/mouse i.c. were transplanted in CD2F1 mice.

Two different P388 sublines resistant to DX were used. The first, P388/DX Johnson subline (Johnson *et al.*, 1978), was maintained by weekly i.p. passages of $10^6$ cells in BD2F1 mice and in experiments $10^5$ cells i.v. and $10^6$ cells subcutaneously and i.p. were transplanted in the same strain of mice.

The second one, the P388 DX Schabel subline, was obtained by repeated exposure to the drug in Dr F. Schabel's laboratory (Southern Research Institute, Birmingham, AL, USA) and maintained in our facilities in BD2F1 mice, given weekly i.p. passages of $10^7$ cells/mouse. The animals were treated 48 h after tumour transplantation with 6 mg $kg^{-1}$ of DX. For experimental studies $10^6$ cells/mouse i.p. or i.v. were transplanted. The L1210 murine leukaemia and its subline resistant to L-PAM, L1210/L-PAM (originally obtained from the NCI, NIH, Bethesda, MD, USA) were maintained by weekly i.p. passages of $10^6$ cells in DBA/2 mice; in the case of L1210/L-PAM, mice were treated weekly with 7.5 mg $kg^{-1}$ L-PAM i.p. For experimental studies, i.p. or i.v. inocula of $10^5$ cells into CD2F1 mice were used. The L1210 subline resistant to cDDP, L1210/cDDP (originally obtained from NCI, NIH, Bethesda, MD, USA) was maintained by weekly i.p. passages of $10^6$ cells/mouse in DBA/2 mice, treated weekly with 5 mg $kg^{-1}$ of cDDP; in experiments, $10^5$ cells/mouse were transplanted i.v. in CD2F1 mice.

*Solid tumours* The Lewis lung carcinoma 3LL ($10^5$ cells/mouse) and the M5076 murine reticulosarcoma ($5 \times 10^5$ cells/mouse) (obtained from the DCT Tumor Repository, NCI, Frederick, MD, USA), were tranplanted i.m. in C57Bl/6 mice to evaluate the activity on primary tumour. The murine mammary ca. (MTV) ($20 \times 10^6$ cells/mouse) from a third generation spontaneous tumour was inoculated s.c. in C3H/He females (Di Marco *et al.*, 1972).

The murine Colon 38 tumour was transplanted s.c. in C57Bl/6 mice using 15–20 mg of tumour brei. MX1 human mammary carcinoma and CX1 human colon carcinoma (NCI, NIH, Bethesda, MD, USA), N592 human small cell lung carcinoma and A549 lung adenocarcinoma (ATCC catalogue), LoVo and LoVo/DX colon carcinoma (Grandi *et al.*, 1986) were transplanted s.c. in athymic mice using 15–20 mg of tumour brei.

### Drug administration

All drug solutions were prepared immediately before use. Treatment was administered i.p., i.v. or orally (by stomach tube) in a volume of 10 ml $kg^{-1}$ of body weight. Treatment schedules are reported in the Results.

### Evaluation of anti-tumour activity and toxicity

In experiments in leukaemia models, drug activity was determined by comparing the median survival time (MST) of the treated group with that of the control group, and results are expressed as %T/C, where:

$$\%T/C = \frac{\text{MST of treated group}}{\text{MST of control group}} \times 100$$

In experiments with solid tumours, primary tumour growth was assessed by caliper measurement, and tumour weight was estimated according to Geran *et al.* (1972). The anti-tumour effect was determined by change of tumour weights of the treated group and that of the control group on a given day.

The percentage of tumour growth inhibition (%TI) was calculated 1 week after the last treatment according to the equation:

$$100 - \frac{\text{median tumour weight of treated group}}{\text{median tumour weight of control group}} \times 100$$

The number of long-term survivors refers to mice surviving at the end of the experiment: >60 days from tumour implant for leukaemias, >120 days from tumour implant for murine solid tumours. For human solid tumours, the tumour-free mice 60 days after tumour implant are considered cured mice.

Toxicity was evaluated on the basis of the gross autopsy findings and the weight loss. In the experiments on solid tumours, tumour-bearing mice were observed for 4 months after the beginning of treatment for evaluation of lethality. Mice are considered to have died of toxicity when death occurred before the controls, or when significant body weight loss and/or spleen and liver size reductions were observed.

### Results

The structure of FCE 23762 is reported in Figure 1. The lipophilicity of the compound was evaluated by means of a direct RP-HPLC (reverse phase-high performance liquid chromatography) method (Facchetti *et al.*, 1991) and lipophilicity is expressed as the capacity factor evaluated at 0% of the organic phase (log $K'_0$) which is the retention index. At pH 7, FCE 23762 (log $K'_0 = 2.768$) is more lipophilic than DX (log $K'_0 = 0.795$).

### Antileukaemic activity

Results obtained by comparing the anti-tumour activity of FCE 23762 and DX on P388 and P388/DX leukaemias are reported in Table I.

Against i.p. implanted P388 leukaemia, FCE 23762 and DX presented equivalent efficacy with a %T/C value of 243 and 290 at the optimal dose of 0.15 mg $kg^{-1}$ and 15 mg $kg^{-1}$ respectively. Against the two ascitic DX-resistant P388 sublines, the compound maintained activity with a %T/C value of 155 and 165, whereas DX was completely ineffective. Equivalent results were observed after i.v. and oral treatment against disseminated P388 and P388/DX leukaemias; FCE 23762 was in fact able to increase the survival time in all three models at the optimal dose of 0.092–0.11 mg $kg^{-1}$ and 0.15 mg $kg^{-1}$ after i.v. and oral administration respectively. In the evaluation of the anti-tumour activity by the oral route, the comparison with DX is not reported. DX is in fact inactive by this route (Barbieri *et al.*, 1987).

Table II demonstrates that the efficacy observed against i.p. or i.v. injected P388/DX leukaemia is maintained when cells are implanted subcutaneously. In fact tumour inhibition values of 90% and 85% were observed with the two tested treatment schedules; this activity was also reflected by a remarkable increase in the survival time. The two schedules utilised appear to be equally effective. This was also observed against disseminated P388/DX leukaemia where different repeated treatment schedules were assayed, obtaining anti-tumour activity equivalent to that observed after single administration (data not shown).

Because of the high lipophilicity of the compound, we investigated the activity of FCE 23762 against intracranially implanted P388 leukaemia (Table III). In this particular model, we utilised BCNU as positive control. The drug given as single i.v. treatment was ineffective in increasing the survival time of mice; this result representing a possible indication that the compound does not cross the blood-brain barrier.

The anti-tumour activity of FCE 23762 was also explored against disseminated L1210 murine leukaemias sensitive and

**Table I** Activity of FCE 23762 and DX against P388 sensitive and resistant to DX (P388/DX) murine leukaemias

| Compound | Tumour site[a] | Treatment schedule | Dose ($mg\,kg^{-1}$) | P388 %T/C[b] | P388 TOX[c] | P388/DX J. %T/C[b] | P388/DX J. TOX[c] | P388/DX S. %T/C[b] | P388/DX S. TOX[c] |
|---|---|---|---|---|---|---|---|---|---|
| FCE 23762 | i.p. | i.p. $d_1$ | 0.15 | 243 | 0/30 | 155 | 0/10 | 165 | 1/10 |
| | | | 0.2 | 148 | 22/40 | 81 | 10/10 | – | – |
| DX | i.p. | i.p. $d_1$ | 10 | 240 | 0/50 | 100 | 0/10 | 110 | 0/10 |
| | | | 15 | 290 | 1/10 | 104 | 2/10 | – | – |
| FCE 23762 | i.v. | i.v. $d_1$ | 0.092 | 250 | 0/10 | 192 | 0/39 | 153 | 0/18 |
| | | | 0.11 | 88 | 10/10 | 208 | 4/49 | 130 | 15/18 |
| | i.v. | os. $d_1$ | 0.12 | 160 | 0/10 | 167 | 0/10 | n.t. | |
| | | | 0.15 | 200 | 0/10 | 175 | 1/20 | n.t. | |
| DX | i.v. | i.v. $d_1$ | 13 | 175 | 1/10 | 95 | 2/20 | 105 | 0/18 |
| | | | 16.9 | 188 | 4/10 | 95 | 3/30 | 99 | 6/18 |

n.t. = not tested. [a]Tumour cells were inoculated at day 0. [b]Median survival time of treated mice/median survival time of controls × 100. [c]Number of toxic deaths/number of mice, evaluated in tumour bearing mice.

**Table II** Activity of FCE 23762 and DX against subcutaneous P388/DX Johnson murine leukaemia with different treatment schedules[a]

| Compound | Route and treatment schedule | Dose ($mg\,kg^{-1}$) | %T/C[b] | %TI[c] | TOX[d] |
|---|---|---|---|---|---|
| FCE 23672 | i.v. $d_{1,8}$ | 0.031 | 111 | 23 | 0/10 |
| | | 0.047 | 118 | 45 | 0/10 |
| | | 0.07 | 170 | 90 | 0/9 |
| DX | i.v. $d_{1,8}$ | 6 | 103 | 32 | 1/9 |
| | | 7.5 | 107 | 38 | 1/10 |
| | | 9 | 96 | 50 | 1/10 |
| FCE 23762 | i.v. $d_{1,4,7,11}$ | 0.025 | 111 | 31 | 0/9 |
| | | 0.0325 | 141 | 29 | 0/10 |
| | | 0.05 | 196 | 85 | 0/10 |
| | | 0.075 | 96 | n.d. | 10/10 |
| DX | i.v. $d_{1,4,7,11}$ | 3 | 103 | 16 | 0/9 |
| | | 4.5 | 111 | 59 | 1/8 |
| | | 6.75 | 107 | 61 | 1/8 |

[a]$10^6$ cells/mouse implanted s.c. in a volume of 0.5 ml at day 0. [b]Median survival time of treated mice/median survival time of controls × 100. [c]The percentage of tumour growth inhibition was calculated in respect to controls on day 11 after tumour cell transplantation (day 0). [d]Number of toxic deaths/number of mice, evaluated in tumour bearing mice.

**Table III** Activity of FCE 23762 and BCNU against intracranially transplanted P388 leukaemia[a]

| Compound | Route and treatment schedule | Dose ($mg\,kg^{-1}$) | %T/C[b] | TOX[c] |
|---|---|---|---|---|
| FCE 23762 | i.v. $d_1$ | 0.09 | 104 | 0/10 |
| | i.v. $d_1$ | 0.11 | 112 | 0/10 |
| | i.v. $d_1$ | 0.13 | 60 | 9/10 |
| BCNU | i.v. $d_1$ | 10 | 180 | 0/10 |
| | i.p. $d_1$ | 20 | >480 | 0/10 |
| | i.p. $d_1$ | 30 | >480 | 0/10 |

[a]$10^4$ cells were implanted intracranially in a volume of 0.02 ml at day 0. [b]Median survival time of treated mice/median survival time of controls × 100. [c]Number of toxic deaths/number of mice, evaluated in tumour bearing mice.

resistant to L-PAM (L1210/L-PAM) and cDDP (L1210/cDDP) (Table IV). The compound was equally effective on the sensitive and cDDP resistant leukaemias, as indicated by the %T/C values of 169 and 164 after i.v. administration, and 150 and 171 after oral administration; on the subline resistant to L-PAM, the compound was also effective, although at a lesser degree. The increase in survival time was in fact of 21% (i.v. route) and of 40% (oral route).

### *Anti-tumour activity against solid murine models*

A parallel evaluation of the activity of FCE 23762 and DX was carried out on four solid murine tumour models. In all models only the results with DX at the optimal dose are reported.

Table V presents results obtained in two i.m. implanted solid tumours. On Lewis lung carcinoma, FCE 23762 was

**Table IV** Activity of FCE 23762 on disseminated L1210, L1210/L-PAM and L1210/cDDP leukaemias

| Cell line[a] | Route and treatment schedule | Dose ($mg\,kg^{-1}$) | %T/C[b] | TOX[c] |
|---|---|---|---|---|
| L1210 | i.v. $d_1$ | 0.092 | 146 | 0/20 |
| | | 0.11 | 169 | 0/18 |
| | os. $d_1$ | 0.16 | 150 | 0/10 |
| | | 0.2 | 183 | 3/20 |
| L1210/L-PAM | i.v. $d_1$ | 0.092 | 121 | 0/20 |
| | | 0.11 | 134 | 4/29 |
| | os. $d_1$ | 0.2 | 140 | 0/20 |
| | | 0.3 | 148 | 7/20 |
| L1210/cDDP | i.v. $d_1$ | 0.092 | 164 | 0/10 |
| | | 0.11 | 114 | 8/20 |
| | os. $d_1$ | 0.2 | 171 | 0/10 |
| | | 0.3 | 100 | 8/10 |

[a]$10^5$ cells were transplanted i.v. at day 0. [b]Median survival time of treated mice/median survival time of controls × 100. [c]Number of toxic deaths/number of mice, evaluated in tumour bearing mice.

marginally effective in reducing tumour growth, as indicated by %TI values of 36 obtained after i.v. administration of $0.1\,mg\,kg^{-1}$ at days 1, 8, 15 and %TI of 40 after oral administration with $0.13\,mg\,kg^{-1}$ every 4 days. On M5076 reticulosarcoma, the compound was as active as DX, both in inhibiting tumour growth (%TI 94) and in increasing the survival time (%T/C 159).

Results obtained testing the activity of FCE 23762 on s.c. implanted MTV mammary carcinoma and Colon 38 models are reported in Table VI.

The compound was as active as DX against MTV carcinoma, as indicated by %TI values of 75 and 90 after i.v. and oral treatment, this last treatment also being effective in increasing the survival time (%T/C 150). Conversely, the compound differs from DX in being inactive against the colon 38 model.

### *Anti-tumour activity against human models*

The activity of FCE 23762 in comparison with DX on a panel of s.c. implanted human tumour models, is reported in Tables VII and VIII. Table VII reports results obtained on a human mammary carcinoma, MX-1 and two lung tumours, N592 (small cell carcinoma) and A549 (adenocarcinoma).

The compound shows better efficacy than DX on MX1 mammary carcinoma, both after i.v. and oral administrations, giving a 99% reduction of tumour growth (%TI) with 90% of cured mice. Better efficacy in comparison with DX was also observed on N592 small cell lung carcinoma (%TI 89 *vs* 55), whereas both compounds were marginally effective on A549 adenocarcinoma (%TI 29 and 31).

Table VIII presents the results obtained testing the activity of FCE 23762 on two human colon models, CX-1 and LoVo, and the derived DX-resistant tumour, LoVo/DX. In the experimental conditions investigated, no anti-tumour activity

**Table V** Activity of FCE 23762 and DX on 3LL and M5076 murine solid tumour models

| *Tumour and site of implant*[a] | *Compound* | *Route and treatment schedule* | *Dose ($mg\ kg^{-1}$)* | *%T/C*[b] | *%TI*[c] | *TOX*[d] |
|---|---|---|---|---|---|---|
| 3LL i.m. | FCE 23762 | i.v. $d_{1,8,15}$ | 0.1 | 133 | 36 | 0/10 |
| | | | 0.13 | 80 | n.d. | 10/10 |
| | | os. $d_{4,8,12,16}$ | 0.13 | 113 | 40 | 0/10 |
| | | | 0.16 | 117 | 60 | 4/10 |
| | DX | i.v. $d_{1,8,15}$ | 7.5 | 172 | 100 | 0/20 |
| M5076 i.m. | FCE 23762 | i.v. $d_{1,8,15}$ | 0.075 | 159 | 94 | 0/10 |
| | | | 0.1 | 66 | n.d. | 10/10 |
| | DX | i.v. $d_{1,8,15}$ | 6 | 143 | 93 | 0/10 |

[a]Tumour cells were inoculated at day 0. [b]Median survival time of treated mice/median survival time of controls × 100. [c]The percentage of tumour growth inhibition was calculated in respect to controls 1 week after tumour cell transplantation (day 0). [d]Number of toxic deaths/number of mice, evaluated in tumour bearing mice.

**Table VI** Activity of FCE 23762 and DX on MTV and on Colon 38 murine solid tumour models

| *Tumour and site of implant*[a] | *Compound* | *Route and treatment schedule* | *Dose ($mg\ kg^{-1}$)* | *%T/C*[b] | *%TI*[c] | *TOX*[d] |
|---|---|---|---|---|---|---|
| MTV s.c. | FCE 23762 | i.v.- q7d × 4 | 0.05 | 138 | 75 | 0/10 |
| | | | 0.075 | 121 | 90 | 4/6 |
| | | os.- q7d × 4 | 0.12 | 150 | 90 | 0/9 |
| | | | 0.155 | 65 | n.d. | 9/9 |
| | DX | i.v.- q7d × 4 | 6 | 124 | 91 | 1/9 |
| Colon 38 s.c. | FCE 23762 | i.v.- q7d × 4 | 0.05 | 90 | 7 | 0/9 |
| | | | 0.075 | 51 | 32 | 3/9 |
| | | os.- q7d × 4 | 0.12 | 86 | 20 | 0/9 |
| | | | 0.15 | 77 | 35 | 3/9 |
| | DX | i.v.- q7d × 4 | 6 | 93 | 82 | 0/9 |

[a]Tumour cells were inoculated at day 0. Treatment was started when the tumour was palpable. [b]Median survival time of treated mice/median survival time of controls × 100. [c]The percentage of tumour growth inhibition was calculated in respect to controls 1 week after tumour cell transplantation (day 0). [d]Number of toxic deaths/number of mice, evaluated in tumour bearing mice.

**Table VII** Antineoplastic activity of FCE 23762 on human solid tumours xenografted in athymic mice

| *Tumour*[a] | *Compound* | *Route and treatment schedule* | *Optimal dose*[b] *($mg\ kg^{-1}$)* | *%TI*[c] | *%Cured mice*[d] |
|---|---|---|---|---|---|
| MX1 | FCE 23762 | i.v.- q7d × 3 | 0.07 | 99 | 19/20 |
| | | os.- q7d × 3 | 0.13 | 99 | 5/7 |
| | DX | i.v.- q7d × 3 | 6.6 | 72 | 0/7 |
| N592 | FCE 23762 | i.v.- q7d × 3 | 0.085 | 89 | 0/8 |
| | DX | i.v.- q7d × 3 | 6 | 55 | 0/8 |
| A549 | FCE 23762 | i.v.- q4d × 4 | 0.045 | 29 | 0/7 |
| | DX | i.v.- q4d × 4 | 4 | 31 | 0/7 |

[a]Tumour fragments (3 $mm^3$) were implanted s.c. by trocar on athymic (nu/nu) mice on day 0. Treatment was started when the tumour was palpable. [b]Optimal dose is defined as the dosage giving the best %TI with toxicity <10%. [c]The percentage of tumour growth inhibition was determined a week after the end of the treatment. [d]All mice which are tumour free 60 days after tumour implant.

**Table VIII** Antineoplastic activity of FCE 23762 and DX on human colon adenocarcinomas xenografted in athymic mice

| *Tumour*[a] | *Compound* | *Route and treatment schedule* | *Optimal dose*[b] *($mg\ kg^{-1}$)* | *%TI*[c] |
|---|---|---|---|---|
| CX-1 | FCE 23762 | i.v.- q4d × 4 | 0.045 | 9 |
| | DX | i.v.- q4d × 4 | 5.2 | 34 |
| LoVo | FCE 23762 | i.v.- q7d × 3 | 0.046 | 33 |
| | | i.v.- q4d × 4 | 0.045 | 43 |
| | | os.- q4d × 6 | 0.08 | 52 |
| | DX | i.v.- q4d × 4 | 4 | 83 |
| LoVo/DX | FCE 23762 | i.v.- q4d × 4 | 0.06 | 37 |
| | | os.- q4d × 6 | 0.1 | 37 |
| | DX | i.v.- q4d × 4 | 5.2 | 12 |

[a]Tumour fragments (3 $mm^3$) were implanted s.c. by trocar on athymic (nu/nu) mice on day 0. Treatment was started when the tumour was palpable. The number of mice for group range from 6 to 9. [b]Optimal dose is defined as the dosage giving the best %TI with toxicity < 10%. [c]The percentage of tumour growth inhibition was determined 1 week after the end of the treatment.

was observed on the CX-1 model, after treatment with FCE 23762 and only marginal activity after treatment with DX. Comparable although borderline values in terms of %TI were observed on LoVo and LoVo/DX tumours when the compound was administered i.v. and orally with different treatment schedules; in these models DX was active only on the LoVo model (%TI 83) and inactive on LoVo/DX model (%TI 12).

In all tested models, the major toxic effects observed after FCE 23762 treatment were body weight loss and organs reduction, in particular spleen and liver, these being the classic toxic effects of anthracyclines.

### Discussion

This report presents the pattern of anti-tumour activity of FCE 23762, a novel derivative of DX bearing the methoxy-morpholinyl group at position 3′ of the sugar moiety.

The high lipophilicity of the molecule confers to FCE 23762 the characteristic of also being effective after oral administration; with this route, the optimal doses were between 1.5 and 2-fold higher than after i.v. administration. In contrast with previous observations on another morpholino derivative MX-2 (Izumoto *et al.*, 1990) which is also highly lipophilic, FCE 23762 is inactive on intracranially implanted P388 leukaemia, this finding pointing to the possibility that the compound does not pass the blood-brain barrier.

Lipophilicity presumably also plays a role in the observed efficacy of FCE 23762 on DX-resistant cells *in vitro* and *in vivo*. In fact, anthracyclines more lipophilic than DX or DNR are generally more active on MDR cells (Facchetti *et al.*, 1991) and are able to reach higher intracellular concentrations. Among these, FCE 23762 was shown to accumulate at high levels in all tested tumour cell lines, both sensitive and expressing the MDR phenotype (Grandi *et al.*, 1990*b*). Another factor to be taken into consideration is, however, the possible lower affinity to p170 of this class of compounds.

The results presented in this report indicate a consistent efficacy of FCE 23762 on DX-resistant P388 leukaemia cells implanted at different sites and with different routes of administration. This lack of cross-resistance to DX is also confirmed on two solid human tumour models, LoVo and LoVo/DX, where treatment with the compound was similarly effective, although at borderline levels of efficacy. Similar results were obtained on a murine fibrosarcoma model UV-2337 sensitive and resistant to DX (Giavazzi, in preparation).

FCE 23762 was also effective on L1210 murine leukaemia and on two L1210 sublines resistant to L-PAM and cDDP, in the latter showing an activity equivalent to that seen on the wild-type line.

On solid tumours models FCE 23762 was able to inhibit tumour growth on different systems, with remarkable efficacy on MTV mammary carcinoma, M5076 murine reticulosarcoma, N592 human small cell lung cancer and MX-1 human mammary carcinoma. In this last model the drug was consistently more effective than DX, with an elevated number of mice surviving tumour-free after $>60$ days.

FCE 23762 differs from most anthracyclines in being activated to a highly potent metabolite(s) when injected *in vivo*. This is suggested by the finding that FCE 23762 is only 3-4 fold more cytotoxic than DX *in vitro*, whereas it is $>80$ fold more potent when administered to mice (Grandi *et al.*, 1990*b*). This contention is reinforced by the recent report of Lau et al. (1991), describing FCE 23762 being metabolised *in vitro* in the presence of human liver microsomes to a highly cytotoxic metabolite(s) able to alkylate DNA. Notwithstanding this possible alkylating activity, however, FCE 23762 maintains anti-tumour efficacy on L1210 leukaemias resistant to cDDP and L-PAM. The structure of the metabolite(s) is under active investigation.

The toxicity observed after treatment with FCE 23762 are those typical of classic anthracyclines with a low therapeutic index at the treatment schedules employed. We are now setting up a sensitive enough analytical method to evaluate the plasma AUC (area under curve) at the therapeutic and toxic doses, with the objective of identifying the best treatment schedules. However, because of its efficacy *in vitro* and *in vivo* on MDR tumour cells and pattern of anti-tumour activity in the tested models, as well as its unusual mode of action, this compound is recommended for clinical testing.

### References

BARBIERI, B., GIULIANI, F.C., BORDONI, T. & 7 others (1987). Chemical and biological characterization of 4′-iodo-4′-deoxydoxorubicin. *Cancer Res.*, **47**, 4001.

BECK, W.T. (1987). The cell biology of multiple drug resistance. *Biochem. Pharmacol.*, **36**, 2879.

BECK, W.T. (1990). Multidrug resistance and its circumvention. *Eur. J. Cancer*, **26**, 513.

COLEY, H.M., TWENTYMAN, P.R. & WORKMAN, P. (1990). 9-Alkyl-morpholinyl anthracyclines in the circumvention of multidrug resistance. *Eur. J. Cancer*, **26**, 665.

DI MARCO, A., LENAZ, L., CASAZZA, A.M. & SCARPINATO, B.M. (1972). Activity of adriamycin (NSC-123127) and daunorubicin (NSC-82151) against mouse mammary carcinoma. *Cancer Chem. Rep.*, **56**, 153.

ENDICOTT, J.A. & LING, V. (1989). The biochemistry of *f*-glycoprotein-mediated multidrug resistance. *Annu. Rev. Biochem.*, **58**, 137.

FACCHETTI, I., GRANDI, M., CUCCHI, P., GERONI, C., PENCO, S. & VIGEVANI, A. (1991). Influence of lipophilicity on cytotoxicity of anthracyclines in LoVo and LoVo/DX human cell lines. *Anti-Cancer Drug Design*, **6**, 385.

GERAN, R.I., GREENBERG, N.H., MACDONALD, M.M., SCHUMAKER, A.M. & ABBOTT, B.J. (1972). Protocols for screening chemical agents and natural products against animal tumors and other biological systems. *Cancer Chem. Rep.*, Part 3, **3**, 1.

GOLDSTEIN, L.J., GALSKI, H., FOJO, A. & 11 others (1989). Expression of a multidrug resistance gene in human cancers. *J. Natl Cancer Inst.*, **81**, 116.

GRANDI, M., GERONI, C. & GIULIANI, F.C. (1986). Isolation and characterization of a human colon adenocarcinoma cell line resistant to doxorubicin. *Br. J. Cancer*, **54**, 515.

GRANDI, M., MARIANI, M., BALLINARI, D. & 7 others (1990*a*). Lack of cross resistance (CR) to certain anthracycline analogs in human leukemic multidrug resistant cells (MDR) expressing either P-glyco-protein (Pgp-MDR) or altered DNA topoisomerase II (at-MDR). *Proc. AACR*, 357 (abstract no. 2118).

GRANDI, M., PEZZONI, G., BALLINARI, D. & 5 others (1990*b*). Novel anthracycline analogs. *Cancer Treat. Rev.*, **17**, 133.

IZUMOTO, S., ARITA, N., HAYAKAWA, T. & 4 others (1990). Effect of MX2, a new morpholino anthracycline, against experimental brain tumors. *Anticancer Res.*, **10**, 735.

JOHNSON, R.K., CHITNIS, M.P., EMBREY, W.M. & GREGORY, E.B. (1978). *In vivo* characteristics of resistance and cross-resistance of an adriamycin-resistant subline of P388 leukemia. *Cancer Treat. Rep.*, **62**, 1535.

KAYE, S.B. (1988). The multidrug resistance phenotype. *Br. J. Cancer*, **58**, 691.

LAU, D.H.M., LEWIS, A.D., DURAN, G.E. & SIKIC, B.I. (1991). The cellular and biochemical pharmacology of the methoxy morpholino derivative of doxorubicin, FCE 23762. *Proc. AACR*, **32**, 332 (abstract no. 1970).

ODAINI, M., ANDERSSON, B.S., MCCREDIE, K.B. & BERAN, M. (1986). Drug sensitivity and cross-resistance of the 4′-(9-acrydinylamino) methanesulfon-m-anisidide-resistant sublines of HL-60 human leukemia. *Cancer Res.*, **46**, 3330.

ROTHENBERG, M. & LING, V. (1989). Multidrug resistance: molecular biology and clinical relevance. *J. Natl. Cancer Inst.*, **81**, 907.

WATANABE, W., KOMESHIMA, N., NAKAJIMA, S. & TSURUO, T. (1988). MX2, a morpholino anthracycline, as a new antitumor agent against drug-sensitive and multidrug-resistant human and murine tumor cells. *Cancer Res.*, **48**, 6653.